# Relationships between perception of black triangles appearance, personality factors and level of education

**DOI:** 10.1038/s41598-024-55855-3

**Published:** 2024-03-07

**Authors:** Mahmoud K. AL-Omiri, Danial Waleed Ahmad Atieh, Abdullah A. Al Nazeh, Salem Almoammar, Saeed Awod Bin Hassan, Abdulkhaliq Ali F. Alshadidi, Lujain Ibrahim N. Aldosari, Ahmad Aljehani, Naji M. Shat, Edward Lynch

**Affiliations:** 1 Department of Prosthodontics, School of Dentistry, University of Jordan, Queen Rania Street, Amman, 11942 Jordan; 2 Department of Prosthodontics, The City of London Dental School, Canada Water, Lower Road, London, UK; 3 Private Practice, Amman, Jordan; 4 Department of Pediatric Dentistry and Orthodontics Sciences, College of Dentistry, King Khalid University, Asir-Abha, Saudi Arabia; 5 Department of Restorative Dental Sciences, College of Dentistry, King Khalid University, Asir-Abha, Saudi Arabia; 6 Department of Allied Dental Health Sciences, College of Medical Applied Sciences, King Khalid University, Asir-Abha, Saudi Arabia; 7 Department of Prosthetic Dental Sciences, College of Dentistry, King Khalid University, Asir-Abha, Saudi Arabia; 8 Dental Department-Periodontics Division, King Fahad Armed Forces Hospital, Jeddah, Saudi Arabia; 9 Department of Prosthodontics, Faculty of Dental and Oral Surgery, University of Palestine, Al-Zahra, Palestine; 10 De Montfort University, Leicester, UK

**Keywords:** Black triangles, Interdental papillae, Gingival embrasure, Appearance, Personality, NEO-FFI, Satisfaction, Perception, Smile, Dental diseases, Restorative dentistry

## Abstract

This analytical cross-sectional study evaluated the perception of black triangles (BT) and examined the relationships between the perception of BT, personality factors, different educational backgrounds and demographic factors. 435 participants were included and divided into four groups: dentists, clinical (4th and 5th year) dental students, pre-clinical (3rd year) dental students, and laypeople. Participants’ perception of the attractiveness of smile profiles of maxillary and mandibular anterior dentition with BT was rated using a ten-point VAS scale with 0 being the least, and 10 being the most attractive smile profile. The personality was assessed using the NEO-FFI personality questionnaire. The smile profile with multiple large BT was rated the least attractive for the maxillary (mean = 3.6) and mandibular (mean = 3.9) tested profiles. The smile profile without BT was rated the most attractive for the maxillary (mean = 9.1) and mandibular (mean = 8.8) tested profiles. The dental professionals perceived the maxillary smile profile with multiple large BT as less attractive than the non-dental participants (t = − 2.715, P = 0.007). Being a male, having dental education, having lower Neuroticism scores, as well as having higher Openness, Agreeableness, Conscientiousness, and Extraversion scores predicted and contributed more towards perceiving various tested smile profiles as more attractive. These findings show that black triangles negatively impacts the perception of smile attractiveness, and that personality traits and having dental education impact the perception of smile attractiveness for smiles with black triangles.

## Introduction

With an orientation focused solely on the teeth, the importance of the gingival component for an esthetic smile is not to be overlooked^1^. Although gingival esthetics still represents a fundamental part of an esthetic smile^2,3^, few studies have assessed the perception of altered gingival esthetics, and a proper understanding of this aspect is required^4–6^. However, perception of esthetics is a complicated dynamic phenomenon affected by multiple dimensions including geographic, demographic and psychological factors, amongst others^7–9^.

When the interdental gingival papilla tissue is lost, a triangular space is formed between the dentition that is known as open gingival embrasures or black triangles^10,11^. This leads to speech issues, esthetic concerns, food impaction^12,13^, as well as hindering of proper plaque control^14^.

Black triangles might have a considerable effect on the attractiveness of the smile, and were perceived as the worst among many studied esthetic factors^5,15,16^. However, some researchers found that laypeople and periodontists appraised the inflamed gingiva as worse than black triangles^6^. Furthermore, the presence of black triangles was rated amongst the top three most disliked esthetic problems^17^. Also, the worst perceived position of the black triangles was between the central incisors^6^.

Additionally, dental professionals might be more critical in their assessment of the smile and dental esthetics than laypeople^4,6,18–26^, and this may be because of the idealism posed by their dental education^9^. Also, dental specialists were more critical of the interdental papillary length than the patients, and considered black triangles as less attractive than non specialists or laypeople^18,20,27^. Also, females and younger patients were more critical of black triangles than males and older patients^28,29^. It follows that how people perceive esthetics is determined by several factors including their social and cultural factors, age, gender and education^27,30^. Besides, personality factors have been found to be associated with several dental conditions and treatments^31–34^.

To the best of our knowledge, the literature contains no studies relating the personality factors to the esthetic perception of black triangles, especially when using comprehensive personality assessment tools. In addition, the literature lacks studies that explore this relationship amongst different study groups including dentists, clinical dental students, preclinical dental students and laypeople. Furthermore, the esthetic perception of black triangles in the mandibular arch has not been investigated in previous studies.

Considering the scarcity of studies on the perception of gingival esthetics, this study was conducted to look into the relationship between the perception of black triangles, personality profiles and the different educational backgrounds. This could add further guidance to better understanding of the factors involved in the perception of black triangles significance.

The aim of the current study was to identify the relationship between the perception of black triangles, personality profiles, educational background and demographic factors (age, gender, marital status, monthly income and living place) among dental students, dentists, and laypeople.

The null hypothesis for this study was that there is no relationship between the perception of black triangles, personality profiles, educational backgrounds and demographic factors.

## Materials and methods

### Study design and population

This observational, cross-sectional descriptive investigation received the ethical approval from the Institutional Review Board (IRB) of the University of Jordan (Reference number: 19-2022-238 dated 17-4-2022). It was carried out between June 2022 and October 2022 in the University of Jordan following the guidelines of the Helsinki Declaration (9th version, 2013). The participants were requested to provide a signed written informed consent before participation.

A non-probability, convenient, and purposive sampling was used in this study. The participants were invited, and then approached and recruited whilst they were attending their clinics (4th and 5th year dental students), laboratories (3rd year pre-clinical dental students), offices (employees) and practices (dentists).

Four hundred and fifty participants were invited to participate in this study, and 435 responded and were recruited (response rate = 96.7%). The study participants comprised 4 groups including 3rd year preclinical dental students, 4th and 5th year clinical dental students, dentists and laypeople.

The participants were included if they did not have a history of debilitating disease or mental disorders, able to comprehend the questionnaire or able to provide a signed informed consent. Also, dentists were included if they were currently registered in the Jordan Dental Association and currently practiced dentistry.

Participants with any history of mental disorders or debilitating disease were excluded. Also, non-registered and/or non practicing dentists were excluded.

### Study instruments and procedures

After screening and inclusion, the participants’ demographic data including age, gender, place of residence, marital status, income, educational background, level of education and experience for dentists were gathered and recorded.

**Figure 1:**
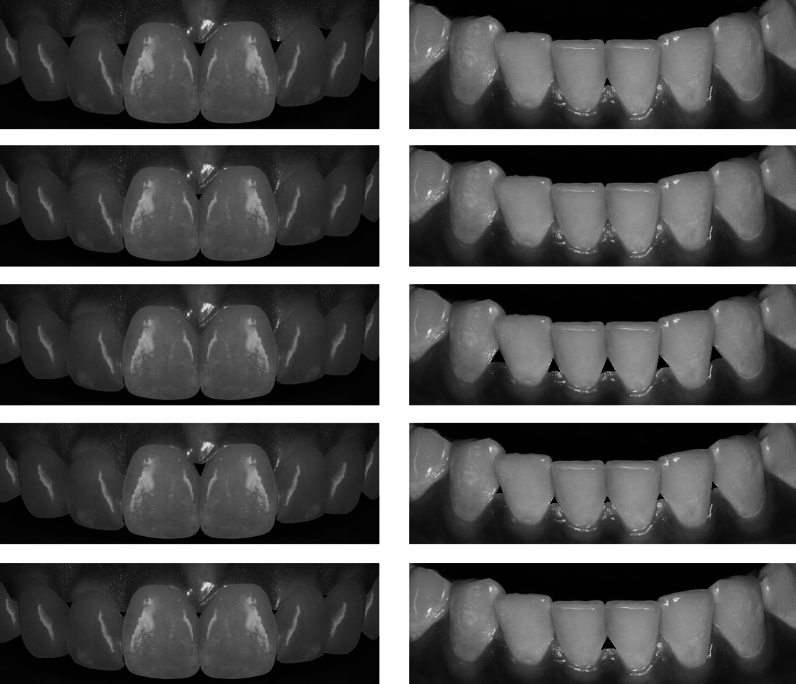
The tested maxillary and mandibular smile images in this study.

Then, the participants’ perception of smiles with black triangles present between the anterior dentition in the maxilla and the mandible was recorded by requesting the participants to rate their perception of the attractiveness of the smile in 10 images (5 maxillary and 5 mandibular) of anterior dentitions (Fig. 1). A visual analogue scale (VAS) from 0 to 10 was utilized for this purpose, where 0 indicates the least attractive and 10 denotes the most attractive.

The rated images were modified photographs of a smile of a female student who provided a signed written informed consent before using the images for this study.

In order to generate the images, the photographs were taken using a digital camera (EOS 90D DSLR, Canon), equipped with a macro lens, and a ring flash with the following settings: f 22, 1:200 speed, ISO 100, a magnification of 1:3, and a flash power of 1/2. The photos were then imported into a digital image processing software (Photoshop CC 2021, Adobe Systems, San Jose, California), and altered so that the nose, chin, lips and the hue were removed to account for the potential confounding variables. A set of teeth template was used for the maxillary arch and adjusted to fit the arch in order to further idealize the teeth. Next, small and large black triangles with rounded edges were made using the polygon tool and their dimensions measured after having resized the images and by comparison with the clinical measurements made generating a small black triangle (1 mm × 1 mm) and a large black triangle (2.5 mm × 1.5 mm). As the tooth dimensions of the maxillary anterior teeth were larger than the mandibular ones, a slightly larger (2.5 mm × 2 mm) black triangle dimension was used between the maxillary teeth. Then, the images were reviewed by 3 experienced prosthodontists for their suitability to use in this study. Finally, the following images (Fig. 1) of the maxillary and mandibular arches were produced and arranged in a random order:

Maxillary dentition images:

Image 1: Smile with multiple large black triangles.

Image 2: Smile with single small central black triangle.

Image 3: Smile without black triangles.

Image 4: Smile with single large central black triangle.

Image 5: Smile with multiple small black triangles.

Mandibular dentition images:

Image 1: Smile with single small central black triangle.

Image 2: Smile without black triangles.

Image 3: Smile with multiple large black triangles.

Image 4: Smile with multiple small black triangles.

Image 5: Smile with single large central black triangle.

Finally, the participants’ personality factors were assessed via the NEO-FFI^35^, which measures the five main personality dimensions: Neuroticism, Extraversion, Openness, Agreeableness and Consciousness. It consists of sixty questions that are answered based on a 5-point Likert scale ranging from ‘strongly agree’ to ‘strongly disagree’. The values of the answers to the NEO-FFI items range from 1 to 5 for each item based on the individual item and the respective dimension. For further information on each individual item and the respective value of its answer, please consult the NEO-FFI manual by Costa and McCrae 1992^35^.

### Study outcome measures and predictors

The main outcome measures for this study were perceptions of black triangles amongst the study sample. The predictors were the participants’ personality scores, level of education, and demographic variables (age, gender, marital status, monthly income and living place).

To assess the reliability of rating the attractiveness of the tested maxillary and mandibular smile images, forty participants (ten from each group) were asked to rank the pictures twice with a one-week interval between the two assessments. In this regard, the intra-class correlation coefficient ranged between 0.896 and 0.989 for the tested maxillary images, and between 0.770 and 0.973 for the tested mandibular images, indicating an adequate reliability.

### Statistical analyses

The statistical work for this investigation was performed using the Statistical Package for Social Sciences (IBM SPSS Statistics v23.0; IBM Corp., USA). The data was tested for normal distribution and the suitable statistical analysis tests were used accordingly. The descriptive statistics was computed for the different variables according to the type of data where the continuous data was described as means, standard errors, standard deviations and confidence intervals, meanwhile the categorical data was described as medians, frequencies, percentages, minimum, maximum and interquartile ranges.

Inferential statistics for parametric variables involved correlation tests between different variables using the Pearson’s r test and the Point biserial correlation (r). It also involved comparison tests for differences between groups using the independent student t-test for two-group comparison and the one-way analysis of variance (ANOVA) test for comparison between more than two groups. The ANOVA was followed by the Scheffe Post hoc test analysis to compare between each two groups. Moreover, hierarchical multivariate linear regression analysis was performed to look for predictors of the perception of black triangles utilizing different independent variables including the demographic and personality factors. The significance level was set as two-tailed with P < 0.05 and a 95% confidence intervals for all the analyses performed.

A priori power analysis was conducted to determine the appropriate sample size using the G*power program (version 3.1.9.7, Heinrich-Heine University, Germany). The ANOVA test was selected in the software, and the following input parameters were entered to the software: an effect size of 0.25 based on a previous study^6^, an α probability error of 0.05, a power (1 − β) of 0.80 and a total of 4 groups. This resulted in a minimum estimated sample size of 180 participants. Extra participants were invited to participate in this study to compensate for participants who might potentially decline, drop out or return incomplete data that cannot be used. Therefore, 450 participants were invited to participate in this study in an attempt to decrease the chance for random error which makes the results more reliable and more representative of the population, especially that this would not cause extra costs and would be easily manageable by the research team. In total, 5 individuals declined to participate and 435 participants responded and were recruited (response rate = 96.7%). Also, none of the 435 recruited participants was excluded later.

## Results

The data from 435 participants (136 males (31.3%) and 299 females (68.7%)) were included and analyzed in this study. The participants’ age ranged between 18 and 78 years old (mean ± SD = 28 ± 10 years old, 95% CI 27–29 years).

Table 1 presents the distribution of the demographic data and variables among the study participants. They were distributed into 4 groups: dentists (n = 110), clinical (4th and 5th year) dental students (n = 110), pre-clinical (3rd year) dental students (n = 104) and laypeople (n = 111) (Table 1).

**Table 1:** The distribution of the categorical demographic data and variables among the study participants.

Categorical data and variables	n (Total = 435)	%
Group		
Dentists		
Total	110	25.3
Females	76	17.5
Males	34	7.8
Clinical (4th and 5th years) dental students		
Total	110	25.3
Females	77	17.7
Males	33	7.6
Pre-clinical (3rd year) dental students		
Total	104	23.9
Females	70	16.1
Males	34	7.8
Laypeople		
Total	111	25.5
Females	76	17.5
Males	35	8.0
Gender		
Male	136	31.3
Female	299	68.7
Marital status		
Single	310	71.3
Married	125	28.7
Educational background		
Dental	324	74.5
Non-dental	111	25.5
Education level (dental)		
Dental student	214	49.2
Bachelors	41	9.4
Higher studies	69	15.9
Education level (non-dental)		
Diploma	37	8.5
Bachelors	59	13.6
Higher studies	15	3.4

### Personality scores amongst the study population

Table 2 demonstrates the distribution of personality traits amongst the total study sample as well as each study group. The mean scores were 20 (SD = 6) for Neuroticism, 28 (SD = 5) for Extraversion, 24 (SD = 5) for Openness, 27 (SD = 4) for Agreeableness and 33 (SD = 7) for Conscientiousness personality factor. Male participants scored lower on Neuroticism among the total study sample (t = − 3.241, P = 0.001) as well as amongst the clinical dental students (t = − 4.255, P < 0.001) and the pre-clinical dental students (t = − 2.399, P = 0.018). Also, the males scored lower on Agreeableness amongst the total study sample (t = − 4.358, P < 0.001) as well as amongst the dentists (t = − 2.494, P = 0.014), the clinical dental students (t = − 3.122, P = 0.002), and the pre-clinical dental students (t = − 2.099, P = 0.038). In addition, the males scored higher on Extraversion within the laypeople group (t = 2.054, P = 0.042).

**Table 2:** Personality traits’ measures of central tendency and dispersions among the study sample.

Personality trait	Descriptive	Total sample	G1 (n = 110)	G2 (n = 110)	G3 (n = 104)	G4 (n = 111)
N	M (SD)	20 (6)	19 (6)	21 (7)	22 (5)	20 (7)
	Variance	42	41	45	30	48
	Min–Max	2–42	2–42	6–40	5–38	6–39
	95% CI	20–21	18–20	20–22	21–23	19–21
E	M (SD)	28 (5)	28 (5)	28 (5)	27 (5)	28 (5)
	Variance	26	24	29	27	24
	Min–Max	13–44	13–44	17–41	14–40	15–40
	95% CI	27–28	27–29	27–29	26–28	27–29
O	M (SD)	24 (5)	24 (5)	25 (5)	24 (5)	24 (5)
	Variance	23	20	24	22	27
	Min–Max	11–43	15–38	11–38	12–34	11–43
	95% CI	24–25	24–25	24–26	23–25	23–25
A	M (SD)	27 (4)	28 (3)	26 (4)	26 (5)	26 (5)
	Variance	18	11	15	22	22
	Min–Max	14–38	18–35	15–37	16–38	14–36
	95% CI	26–27	27–28	26–27	25–27	25–27
C	M (SD)	33 (7)	33 (6)	32 (7)	31 (6)	35 (7)
	Variance	43	32	46	39	50
	Min–Max	9–48	9–47	14–46	19–48	17–48
	95% CI	32–33	32–34	31–33	29–32	33–36

*G1* dentists, *G2* clinical (4th and 5th year) dental students, *G3* pre-clinical (3rd year) dental students, *G4* laypeople, *N* neuroticism, *E* extraversion, *O* openness, *A* agreeableness, *C* conscientiousness, *n* number of participants, *M* mean score, *SD* standard deviation, *Min* minimum, *Max* maximum, *CI* confidence intervals.

### Perception of the attractiveness of the tested smile images amongst the study population

Table 3 presents the descriptive statistics and group differences of the participants’ perceptions and rankings (scale scores) of the attractiveness of the tested maxillary and mandibular smile images amongst the study population. The highest mean attractiveness score was recorded for the maxillary (9.1 ± 1.4) and mandibular (8.8 ± 1.5) images with no black triangles, whilst the lowest mean attractiveness scores were recorded for the maxillary (3.6 ± 2.2) and mandibular (3.9 ± 2.2) images with multiple large black triangles. Significantly different perception of smile attractiveness between groups was reported for maxillary images with a single small central black triangle and multiple large black triangles (P < 0.05, Table 3). Further comparisons using Scheffe Post hoc test revealed that pre-clinical (3rd year) dental students perceived the maxillary smile images with single small central black triangle as less attractive than the dentists (mean difference = − 0.675, P = 0.036) and the laymen (mean difference = − 0.853, P = 0.004). Additionally, the clinical (fourth and fifth year) dental students perceived the maxillary smile images with multiple large black triangles as less attractive than the laypeople (mean difference = − 0.944, P = 0.018).

**Table 3:** Descriptive statistics of the participants’ perception and ranking scores for the tested maxillary smile profiles among the study sample (total n = 435).

Image	Descriptive	Total sample	G1 (n = 110)	G2 (n = 110)	G3 (n = 104)	G4 (n = 111)
Maxillary smiles						
No BT	M (SD)	9.1 (1.4)	9.2 (1.1)	9.1 (1.4)	8.9 (1.5)	9.1 (1.7)
	Variance	2.1	1.3	2.0	2.2	2.8
	Min–Max	1.0–10.0	2.0–10.0	3.0–10.0	2.0–10.0	1.0–10.0
	95% CI	8.9–9.2	9.0–9.4	8.8–9.3	8.7–9.2	8.8–9.4
	Group difference	ANOVA: F = 0.521, P = 0.668				
Single small central BT	M (SD)	7.1 (1.7)	7.3 (1.6)	7.0 (1.6)	6.6 (1.9)	7.5 (1,7)
	Variance	2.9	2.5	2.6	3.5	2.8
	Min–Max	0.0–10.0	1.0–10.0	0.0–10.0	0.0–10.0	2.0–10.0
	95% CI	6.9–7.3	7.0–7.6	6.7–7.3	6.3–7.0	7.2–7.8
	Group difference	ANOVA: F = 5.216, P = 0.002				
Single large central BT	M (SD)	6.4 (1.7)	6.5 (1.6)	6.1 (1.7)	6.4 (1.7)	6.7 (1.7)
	Variance	2.8	2.7	2.7	2.9	2.8
	Min–Max	0.0–10.0	0.0–10.0	1.0–9.0	2.0–10.0	0.0–10.0
	95% CI	6.3–6.6	6.1–6.8	5.8–6.5	6.1–6.7	6.4–7.0
	Group difference	ANOVA: F = 2.190, P = 0.089				
Multiple small BT	M (SD)	5.9 (2.0)	5.8 (1.8)	5.7 (1.9)	5.9 (2.1)	6.1 (2.3)
	Variance	4.1	3.3	3.6	4.5	5.2
	Min–Max	0.0–10.0	0.0–10.0	0.0–10.0	1.0–10.0	0.0–10.0
	95% CI	5.7–6.1	5.5–6.2	5.3–6.0	5.5–6.3	5.6–6.5
	Group difference	ANOVA: F = 0.769, P = 0.512				
Multiple large BT	M (SD)	3.6 (2.2)	3.7 (2.0)	3.2 (2.2)	3.3 (2.3)	4.1 (4.6)
	Variance	4.9	4.2	4.8	5.3	4.9
	Min–Max	0.0–10.0	0.0–8.0	0.0–8.0	0.0–10.0	0.0–9.0
	95% CI	3.4–3.8	3.3–4.1	2.8–3.6	2.9–3.8	3.7–4.6
	Group difference	ANOVA: F = 4.100, P = 0.007				
Mandibular smiles						
No BT	M (SD)	8.8 (1.5)	9.1 (1.0)	9.0 (1.4)	8.5 (1.6)	8.7 (1.8)
	Variance	2.3	1.0	2.0	2.6	3.4
	Min–Max	0.0–10.0	4.0–10.0	0.0–10.0	0.0–10.0	1.0–10.0
	95% CI	8.7–9.0	8.9–9.3	8.7–9.3	8.2–8.8	8.3–9.0
	Group difference	ANOVA: F = 3.685, P = 0.012				
Single small central BT	M (SD)	7.2 (1.6)	7.5 (1.4)	7.3 (1.4)	6.8 (1.7)	7.2 (1.8)
	Variance	2.5	2.0	2.0	2.8	3.2
	Min–Max	0.0–10.0	2.0–10.0	3.0–10.0	0.0–10.0	0.0–10.0
	95% CI	7.1–7.4	7.3–7.8	7.0–7.5	6.5–7.2	6.9–7.6
	Group difference	ANOVA: F = 3.631, P = 0.013				
Single large central BT	M (SD)	6.0 (1.8)	6.2 (1.7)	5.9 (1.8)	5.7 (1.8)	6.3 (1.8)
	Variance	3.3	3.0	3.2	3.4	3.4
	Min–Max	0.0–10.0	1.0–9.0	1.0–10.0	0.0–10.0	0.0–10.0
	95% CI	5.9–6.2	5.9–6.6	5.5–6.2	5.3–6.0	6.0–6.7
	Group difference	ANOVA: F = 2.889, P = 0.035				
Multiple small BT	M (SD)	5.6 (2.0)	5.8 (1.9)	5.5 (1.8)	5.5 (2.2)	5.5 (2.0)
	Variance	3.9	3.5	3.3	4.8	4.1
	Min–Max	0.0–10.0	0.0–10.0	0.0–10.0	0.0–10.0	0.0–9.0
	95% CI	5.4–5.8	5.5–6.2	5.2–5.9	5.1–6.0	5.1–5.9
	Group difference	ANOVA: F = 0.671, P = 0.570				
Multiple large BT	M (SD)	3.9 (2.2)	4.2 (1.9)	3.7 (2.1)	3.8 (2.3)	4.1 (2.3)
	Variance	4.7	3.6	4.6	5.2	5.3
	Min–Max	0.0–10.0	0.0–8.0	0.0–9.0	0.0–10.0	0.0–10.0
	95% CI	3.7–4.1	3.8–4.5	3.3–4.1	3.4–4.2	3.6–4.5
	Group difference	ANOVA: F = 1.192, P = 0.313				

*BT* black triangle, *G1* dentists, *G2* clinical (4th and 5th year) dental students, *G3* pre-clinical (3rd year) dental students, *G4* laypeople, *M* mean score, *SD* standard deviation, *Min* minimum, *Max* maximum, *CI* confidence intervals, *ANOVA* Analysis of variance test, *F* F statistic, *P* probability value using ANOVA.

Furthermore, significantly different perception of smile attractiveness between groups was reported for mandibular images with no black triangle, single small central black triangle, and mandibular single large central black triangle (P < 0.05, Table 3). Further comparisons using Scheffe Post hoc test revealed that dentists perceived the mandibular smile images with no black triangle (mean difference = 0.617, P = 0.031) and the mandibular smile images with a single small central black triangle (mean difference = 0.709, P = 0.014) as more attractive than the pre-clinical (3rd year) dental students.

Table 4 shows the difference in the perception of smile attractiveness based on having dental and non-dental educational backgrounds as well as based on gender among the study sample. The male participants perceived the smiles with an maxillary single small central black triangle, maxillary single large central black triangle, maxillary multiple large black triangles, mandibular multiple small black triangles and mandibular multiple large black triangles as more attractive than females (P < 0.05, Table 4).

**Table 4:** Differences in the perception of smile profiles attractiveness based on gender as well as being a dental or non-dental participant (total n = 435).

Smile profiles	Dental vs. non-dental	Descriptive		t-test		Male vs female	Descriptive		t-test	
		Mean	SD	t	P		Mean	SD	t	P
Maxillary smiles										
No BT	Dental	9.05	1.35	− 0.454	0.650	Male	9.10	1.30	0.229	0.819
	Non-dental	9.12	1.63			Female	9.07	1.49		
Single small central BT	Dental	6.97	1.71	− 2.714	0.007	Male	7.46	1.36	2.982	0.003
	Non-dental	7.46	1.64			Female	6.94	1.82		
Single large central BT	Dental	6.33	1.65	− 2.084	0.038	Male	6.71	1.40	2.359	0.019
	Non-dental	6.70	1.68			Female	6.31	1.77		
Multiple small BT	Dental	5.77	1.93	− 1.589	0.113	Male	6.05	1.86	1.231	0.219
	Non-dental	6.12	2.25			Female	5.79	2.11		
Multiple large BT	Dental	3.43	2.18	− 2.715	0.007	Male	4.01	2.30	3.130	0.002
	Non-dental	4.08	2.24			Female	3.39	2.15		
Mandibular smiles										
No BT	Dental	8.88	1.40	1.126	0.261	Male	8.80	1.34	− 0.337	0.736
	Non-dental	8.70	1.80			Female	8.86	1.60		
Single small central BT	Dental	7.21	1.52	− 0.126	0.900	Male	7.15	1.54	0.554	0.580
	Non-dental	7.23	1.76			Female	7.25	1.62		
Single large central BT	Dental	5.90	1.80	− 2.323	0.021	Male	6.25	1.74	1.769	0.078
	Non-dental	6.35	1.83			Female	5.92	1.85		
Multiple small BT	Dental	5.60	1.96	0.103	0.918	Male	5.93	1.82	2.359	0.019
	Non-dental	5.58	2.01			Female	5.45	2.03		
Multiple large BT	Dental	3.89	2.10	− 0.801	0.423	Male	4.45	2.16	3.350	0.001
	Non-dental	4.07	2.29			Female	3.71	2.12		

*BT* black triangle, *M* mean, *SD* standard deviation, *t* t-statistic, *P* probability value using independent t-test.

The participants with dental educational backgrounds perceived the smiles with an maxillary single small central black triangle, maxillary single large central black triangle, the maxillary multiple large black triangles and mandibular single large central black triangle as less attractive than the participants with non-dental educational background (P < 0.05, Table 4).

Amongst the total study sample, older participants perceived the smiles with an maxillary single small central black triangle (r = 0.145, P = 0.002), maxillary single large central black triangle (r = 0.162, P = 0.001), maxillary multiple small black triangles (r = 0.098, P = 0.042), maxillary multiple large black triangles (r = 0.156, P = 0.001), mandibular single small central black triangle (r = 0.122, P = 0.011), mandibular single large central black triangle (r = 0.154, P = 0.001) and mandibular multiple large black triangles (r = 0.116, P = 0.016) as more attractive than younger participants.

### Relationship between personality scores and perception of smile profiles

Correlations between participants’ personality scores and the ranking of smile profile attractiveness amongst the total study sample showed that higher scores of Conscientiousness, Agreeableness, and Openness were associated with better perception of the maxillary smile profile without black triangles and rating it as more attractive (P < 0.05, Table 5). Also, higher Openness scores were associated with perceiving the mandibular smile profile with the single small central black triangle as more attractive (r = 0.105, P = 0.028). Moreover, higher Conscientiousness scores were associated with ranking the smile profiles with the maxillary single small central black triangle, maxillary single large central black triangle, mandibular single small central black triangle, and mandibular single large central black triangle as more attractive (P < 0.05, Table 5). Further significant correlations between personality scores and the ranking of attractiveness were found within each group (P < 0.05, Table 5).

**Table 5:** The correlations between participants’ personality scores and the ranking of the tested maxillary smile profiles attractiveness among the study sample (total n = 435).

Smile profile attractiveness	Per-trait	r (P)				
		Total sample	G1 (n = 110)	G2 (n = 110)	G3 (n = 104)	G4 (n = 111)
Maxillary smiles						
No BT	N	− 0.076 (0.114)	− 0.073 (0.449)	− 0.016 (0.866)	− 0.064 (0.520)	− 0.108 (0.258)
	E	− 0.017 (0.722)	0.058 (0.549)	− 0.077 (0.423)	0.021 (0.831)	− 0.053 (0.578)
	O	0.109 (0.022)	0.238 (0.012)	0.140 (0.144)	0.102 (0.302)	0.014 (0.882)
	A	0.116 (0.015)	− 0.073 (0.446)	− 0.042 (0.665)	0.284 (0.003)	0.162 (0.090)
	C	0.105 (0.028)	0.251 (0.008)	− 0.069 (0.474)	0.125 (0.207)	0.129 (0.176)
Single small central BT	N	− 0.084 (0.081)	0.030 (0.753)	0.017 (0.864)	− 0.279 (0.004)	− 0.032 (0.742)
	E	− 0.013 (0.785)	− 0.046 (0.635)	0.035 (0.720)	0.047 (0.637)	− 0.123 (0.200)
	O	0.017 (0.729)	0.060 (0.536)	− 0.103 (0.283)	0.108 (0.276)	− 0.006 (0.949)
	A	0.092 (0.056)	− 0.124 (0.198)	0.059 (0.539)	0.254 (0.009)	0.069 (0.469)
	C	0.109 (0.022)	0.150 (0.117)	0.040 (0.682)	0.204 (0.038)	− 0.081 (0.399)
Single large central BT	N	− 0.043 (0.371)	0.004 (0.965)	0.047 (0.628)	− 0.099 (0.316)	− 0.107 (0.263)
	E	− 0.036 (0.453)	− 0.080 (0.407)	− 0.128 (0.181)	0.129 (0.192)	− 0.058 (0.543)
	O	0.000 (0.992)	− 0.020 (0.838)	− 0.116 (0.226)	0.097 (0.326)	0.070 (0.468)
	A	0.047 (0.333)	− 0.090 (0.349)	0.030 (0.757)	0.165 (0.094)	0.049 (0.613)
	C	0.140 (0.003)	0.190 (0.047)	− 0.024 (0.802)	0.241 (0.014)	0.121 (0.205)
Multiple small BT	N	− 0.030 (0.539)	− 0.005 (0.961)	− 0.041 (0.669)	− 0.161 (0.103)	0.050 (0.599)
	E	0.039 (0.417)	0.018 (0.851)	− 0.033 (0.736)	0.265 (0.006)	− 0.083 (0.384)
	O	− 0.044 (0.358)	0.060 (0.534)	− 0.225 (0.018)	0.014 (0.889)	− 0.004 (0.967)
	A	0.040 (0.410)	− 0.004 (0.970)	0.032 (0.744)	0.085 (0.388)	0.041 (0.670)
	C	0.022 (0.649)	0.146 (0.128)	− 0.135 (0.158)	0.118 (0.233)	− 0.029 (0.765)
Multiple large BT	N	− 0.044 (0.360)	− 0.005 (0.956)	− 0.150 (0.118)	− 0.166 (0.092)	0.169 (0.077)
	E	0.002 (0.961)	− 0.087 (0.369)	− 0.016 (0.865)	0.155 (0.116)	− 0.058 (0.544)
	O	− 0.054 (0.262)	− 0.053 (0.580)	− 0.279 (0.003)	0.097 (0.328)	0.039 (0.686)
	A	− 0.033 (0.494)	− 0.185 (0.054)	0.075 (0.434)	− 0.079 (0.425)	0.016 (0.866)
	C	0.005 (0.910)	0.158 (0.099)	− 0.140 (0.145)	0.101 (0.306)	− 0.174 (0.067)
Mandibular smiles						
No BT	N	− 0.052 (0.282)	− 0.028 (0.772)	0.000 (0.998)	0.056 (0.572)	− 0.119 (0.213)
	E	0.008 (0.863)	0.041 (0.667)	− 0.102 (0.290)	0.077 (0.439)	0.007 (0.943)
	O	0.086 (0.075)	0.103 (0.286)	0.192 (0.044)	0.006 (0.955)	0.034 (0.727)
	A	0.080 (0.097)	− 0.007 (0.939)	− 0.118 (0.219)	0.270 (0.005)	0.037 (0.703)
	C	0.051 (0.293)	− 0.005 (0.962)	0.000 (0.997)	− 0.038 (0.703)	0.152 (0.112)
Single small central BT	N	0.033 (0.491)	0.038 (0.695)	0.093 (0.332)	− 0.027 (0.788)	0.109 (0.256)
	E	0.014 (0.776)	− 0.025 (0.795)	0.025 (0.796)	0.201 (0.042)	− 0.158 (0.098)
	O	0.005 (0.921)	− 0.097 (0.312)	− 0.017 (0.863)	0.055 (0.582)	0.023 (0.813)
	A	0.105 (0.028)	− 0.087 (0.366)	− 0.051 (0.595)	0.283 (0.004)	0.111 (0.247)
	C	0.095 (0.048)	0.074 (0.439)	0.035 (0.715)	0.157 (0.112)	0.046 (0.633)
Single large central BT	N	− 0.017 (0.722)	0.019 (0.846)	0.060 (0.532)	− 0.090 (0.366)	0.004 (0.970)
	E	− 0.040 (0.409)	− 0.090 (0.349)	− 0.120 (0.213)	0.180 (0.068)	− 0.140 (0.142)
	O	− 0.012 (0.807)	− 0.077 (0.425)	− 0.130 (0.177)	0.150 (0.128)	0.009 (0.927)
	A	0.078 (0.106)	0.027 (0.781)	0.033 (0.733)	0.231 (0.018)	− 0.015 (0.873)
	C	0.108 (0.024)	0.077 (0.427)	− 0.032 (0.736)	0.191 (0.052)	0.097 (0.313)
Multiple small BT	N	− 0.052 (0.279)	0.050 (0.602)	− 0.072 (0.458)	− 0.158 (0.110)	− 0.012 (0.903)
	E	0.063 (0.187)	− 0.005 (0.955)	0.072 (0.454)	0.184 (0.062)	− 0.013 (0.889)
	O	− 0.052 (0.276)	0.071 (0.464)	− 0.211 (0.027)	− 0.071 (0.474)	− 0.009 (0.929)
	A	0.055 (0.256)	− 0.022 (0.823)	0.021 (0.824)	0.222 (0.023)	− 0.073 (0.447)
	C	0.060 (0.215)	0.130 (0.176)	0.022 (0.818)	0.141 (0.154)	− 0.033 (0.728)
Multiple large BT	N	− 0.028 (0.557)	− 0.042 (0.667)	− 0.092 (0.337)	− 0.117 (0.239)	0.144 (0.131)
	E	0.001 (0.988)	− 0.041 (0.671)	− 0.021 (0.828)	0.155 (0.116)	− 0.100 (0.299)
	O	− 0.037 (0.436)	0.011 (0.910)	− 0.207 (0.030)	0.136 (0.169)	− 0.065 (0.498)
	A	− 0.002 (0.960)	− 0.010 (0.920)	− 0.088 (0.360)	0.110 (0.265)	− 0.062 (0.515)
	C	0.018 (0.713)	0.001 (0.996)	− 0.035 (0.713)	0.150 (0.129)	− 0.081 (0.398)

*Per-trait* personality trait, *BT* black triangle, *G1* dentists, *G2* clinical (4th and 5th year) dental students, *G3* pre-clinical (3rd year) dental students, *G4* laypeople, *N* neuroticism, *E* extraversion, *O* openness, *A* agreeableness, *C* conscientiousness, *r* Pearson’s correlations coefficient, *P* probability value using Pearson’s correlations test.

Multiple two-step hierarchical regression analyses were performed to examine the prediction power of personality factors on the perception of smile attractiveness for each tested smile profile whilst controlling for the selected demographic characteristics (age, gender, marital status, city, monthly income, dental/non-dental and group). The results demonstrated that being a male, having dental education, having lower Neuroticism scores, as well as having higher Openness, Agreeableness, Conscientiousness, and Extraversion scores predicted and contributed more towards perceiving various tested smile profiles as more attractive (Table 6).

**Table 6:** Hierarchical regression analysis to predict perception of smile attractiveness ratings utilizing personality scores and demographic variables among the total study sample as well as among each group (total n = 435).

Dependent variable	Group	Predictors*	R^2^	R^2^ change	B	β	P	95% CI for B	
								Lower bound	Upper bound
Maxillary smiles									
No BT	Total	Openness	0.054	0.036	0.036	0.121	0.013	0.008	0.064
	G3	Agreeableness	0.111	0.103	0.099	0.319	0.007	0.028	0.170
	G1	Openness	0.207	0.145	0.071	0.281	0.004	0.023	0.119
		Conscientiousness			0.041	0.203	0.041	0.002	0.080
Single small central BT	Total	Gender	0.083	0.019	− 0.638	− 0.173	0.001	− 0.995	− 0.280
		Dental/non-dental			0.919	0.239	0.005	0.274	1.563
		Group			− 0.259	− 0.171	0.032	− 0.495	− 0.023
		Agreeableness			0.046	0.113	0.027	0.005	0.087
	G2	Gender	0.096	0.040	− 0.945	− 0.272	0.014	− 1.699	− 0.192
	G3	Gender	0.187	0.139	− 0.811	− 0.204	0.047	− 1.611	− 0.010
		Agreeableness			0.105	0.266	0.018	0.019	0.192
	G4	Income	0.162	0.054	0.001	0.339	0.001	0.000	0.001
Single large central BT	Total	Gender	0.062	0.017	− 0.471	− 0.131	0.009	− 0.825	− 0.118
		Conscientiousness			0.029	0.115	0.032	0.003	0.056
	G2	Gender	0.113	0.056	− 0.874	− 0.115	0.026	− 1.643	− 0.105
Multiple small BT	G2	Openness	0.104	0.065	− 0.078	− 0.199	0.047	− 0.154	− 0.001
	G3	Extraversion	0.098	0.085	0.110	0.267	0.017	0.021	0.200
Multiple large BT	Total	Gender	0.071	0.004	− 0.732	− 0.153	0.002	− 1.201	− 0.264
	G2	Openness	0.117	0.102	− 0.108	− 0.238	0.017	− 0.196	− 0.019
	G3	Gender	0.128	0.044	− 1.037	− 0.212	0.047	− 2.058	− 0.016
Mandibular smiles									
No BT	Total	Group	0.034	0.010	− 0.255	− 0.189	0.021	− 0.470	− 0.039
	G2	Openness	0.086	0.055	0.063	0.216	0.033	0.005	0.122
	G3	Agreeableness	0.174	0.127	0.142	0.038	< 0.001	0.066	0.217
Single small central BT	Total	Group	0.055	0.020	− 0.266	− 0.188	0.020	− 0.489	− 0.042
		Neuroticism			0.025	0.104	0.046	0.000	0.050
	G3	Agreeableness	0.121	0.119	0.107	0.303	0.009	0.027	0.188
Single large central BT	Total	Gender	0.060	0.018	− 0.499	− 0.127	0.011	− 0.886	− 0.113
		Dental/non-dental			0.700	0.171	0.049	0.004	1.395
	G3	Agreeableness	0.144	0.130	0.113	0.291	0.012	0.026	0.201
Multiple small BT	Total	Gender	0.040	0.012	− 0.560	− 0.131	0.010	− 0.984	− 0.136
	G2	Gender	0.135	0.054	− 1.147	− 0.290	0.008	− 1.985	− 0.309
		Openness			− 0.080	− 0.215	0.030	− 0.152	− 0.008
	G3	Gender	0.141	0.105	− 1.198	− 0.257	0.015	− 2.159	− 0.236
		Agreeableness			0.132	0.285	0.014	0.028	0.236
	G4	Age	0.087	0.019	0.069	0.407	0.005	0.022	0.116
Multiple large BT	Total	Gender	0.042	0.002	− 0.789	− 0.170	0.001	− 1.252	− 0.327
	G2	Openness	0.102	0.042	− 0.091	− 0.207	0.040	− 0.177	− 0.004
	G3	Gender	0.140	0.064	− 1.386	− 0.286	0.007	− 2.390	− 0.383

*BT* black triangle, *Total* total study sample, *G1* dentists, *G2* clinical (4th and 5th year) dental students, *G3* pre-clinical (3rd year) dental students, *G4* laypeople, *R*^*2*^ coefficient of determination, *B* unstandardized coefficient beta, *β* standardized coefficient beta, *P* two tailed probability value, *CI* confidence intervals.

*Significant predictors in final models are presented; the group, age, gender, dental background, and income were included in the first block of the regression model, while the personality scores were included in the second block.

## Discussion

The results of this study revealed the existence of an association between the perception of black triangles and personality traits, educational backgrounds and different demographic variables. Consequently, the null hypothesis was rejected.

The tested close-up smile profiles in this study were edited to crop the nose, chin, and to conceal the gender of the participant in order to account for the confounding variables^6,27,36,37^. Black and white images were used to avoid the confounding effects of tooth shade and soft tissue texture and colour on perception following previous recommendations^6,38^. Furthermore, the tested images were arranged in a random order to account for potential bias during the ratings^39^.

Besides, a 10-point visual analogue scale (VAS) was used to assess the perception, which is considered a simple and reliable method for assessment^40^. Adequate level of reliability was shown for all ratings of the tested smile profile images in this study.

The NEO-FFI personality test was used in this study because it is comprehensive, simple, valid, reliable and has been used in previous studies among the Jordanian population^31,33–35,41–48^.

In this study, females exhibited higher Neuroticism scores than males, which is in accordance with the results of previous studies^45,49^. This might owe to that the lifestyle of women is more stressful than men. Also, females scored higher Agreeableness scores, which may be because of social factors and the nature of women being more sympathetic and caring than men.

Concerning the maxillary smile profiles, the highest rating was given to the smile profile without black triangles, which agrees with previous studies where participants rated the smile profiles without black triangles as most pleasing^4,6,18,28^. A gradual decrease in the mean scores was found as the size and number of black triangles increased, with the maxillary smile profile with multiple large black triangles receiving the lowest scoring, and this supports other studies that showed lower rating of the photos as the size of the black triangle increases^4,18,28^, and as the number of black triangles increases^5,6^.

Similarly, the most attractive mandibular smile profile was the one without black triangles; meanwhile, the least attractive was the one with multiple large black triangles. Previous studies concerned with black triangles on the mandibular arch could not be identified in the literature, so those results are novel. However, results from the studies on the maxillary arch could be referred to as they showed similar findings^4–6,18,28^.

The study findings showed that female participants assigned lower attractiveness ratings than males. This agrees with previous research that found women to be more judgmental in their evaluation of black triangles^29,37^. This could be because females are more concerned about esthetics than males^45^. However, this does not agree with the results of other studies investigating different esthetic factors^6,50–52^. Variations in study methodologies, tested parameters, psychological, cultural, social and racial factors might explain this difference.

Also, older participants assigned higher attractiveness ratings than younger ones. This may be explained by that younger individuals may seek better appearance as compared to older ones. Multiple studies showed that older individuals were less critical regarding to esthetics^29,32,49,53^. Nonetheless, this was not shown in other studies^6,52^, which probably owe to differences in study methodologies and tested parameters as well as social, psychological, cultural and racial factors.

Participants with a dental background perceived the black triangles as less attractive. This stem from the fact that they were more likely to be exposed to black triangles as an esthetic problem during their dental education and practice, and hence, they are stricter in judging and better at spotting them. This agrees with other studies showing that individuals with a dental background were less lenient in judging different esthetic parameters than laypeople^6,18–20,22–26,38,54–56^. However, this opposes other studies that could not find any difference^27,57–61^.

Differences in psychological, cultural, social and racial factors could account for this contrast, as well as differences in the tested parameters and methodologies adopted during these studies.

The findings of the study showed that pre-clinical (3rd year) dental students were more strict than dentists in their assessment of the mandibular smile profiles, which could be because dentists have better experience and could have better recognized that the mandibular teeth may not show up upon smiling, as opposed to the pre-clinical dental students, whom may not have been taught about this yet. In addition, no differences could be found between the pre-clinical and the clinical dental students, which opposes the study by Alhammadi and colleagues, that found these differences^62^. This could be due to that pre-clinical dental students were limited to 3rd year students in the current study as well as variations in personality and demographic factors.

In this study, Neuroticism was associated with inferior perception, while Openness, Agreeableness, Extraversion and Conscientiousness were associated with a better perception of the attractiveness of the tested smile profiles.

This might be explained as neurotic individuals are characterized by higher expectations and stricter judgment of the appearance^49,53^. Nevertheless, open individuals are open-minded, insightful, inquisitive, and tend to seek new experiences, making them more likely to score higher even for the smile profiles with esthetic problems. Similarly, agreeable individuals are empathetic, compassionate, and more tolerant and accepting of imperfections^49^. Moreover, conscious patients are focused, systematic and meticulous^45^, readily report any improvements and value even the small details^33^, which might clarify why they had enhanced perception. Furthermore, extraverted individuals are positive and optimistic, and so would likely not be affected by minor esthetic issues^49,53^.

The above findings go along with the findings of previous studies that tested other parameters and treatments^34,42,43,45,46,49,63–65^. In contrast, other researchers could not find a relationship between personality and the perception of esthetics^18^, which might be due to testing other esthetic parameters than black triangles and using an incomprehensive personality test.

To the investigators’ best knowledge, this is the first study looking at the relationship between personality profiles and the perception of smile profiles with black triangles. The discussion above indicates that different personality traits could anticipate and contributed towards patients’ expectations. Therefore, it may be advisable to consider patients’ personality during clinical assessment as this might help to reach a more predictable treatment outcome.

In the present study, racial, social and cultural factors might impact the results. Besides, the unbalanced beyond control sample distribution in certain aspects like age, gender, and marital status could have influenced the results. However, the hierarchical regression analysis accounted for the confounding effects of demographic factors on the relationship between personality factors and the perception of tested smile profiles. Also, only tooth images were rated to avoid the distracters that might affect the ratings of the tested images following previous recommendations^6,27,36–39^. This, especially for the laypeople, could represent an unfamiliar image of the smile and might drew more attention to the black triangles. Therefore, rating full face smile images might have been more realistic and further studies are required in this regard.

More studies are advisable on different populations using larger samples to highlight the possible effects of cultural, social and racial factors on the relationships between the perception of black triangles (especially in the mandibular arch), personality profiles, and education.

## Conclusions

Within the limitations of the present study, it was concluded that for both the maxillary and mandibular smile profiles, the images without black triangles represented the most attractive smile profiles, while the images with multiple large black triangles were the least attractive. Also, dental participants, females and younger participants were more critical and assigned lower perception scores for black triangles. Also, personality traits could predict and contributed positively (Openness, Agreeableness, Conscientiousness, and Extraversion) or negatively (Neuroticism) towards the perception of black triangles.

## Data Availability

Data generated and analysed during this study are available from the corresponding author upon request to the following email: alomirim@yahoo.co.uk.
